# Secure and Usable User-in-a-Context Continuous Authentication in Smartphones Leveraging Non-Assisted Sensors

**DOI:** 10.3390/s18041219

**Published:** 2018-04-16

**Authors:** Jose Maria de Fuentes, Lorena Gonzalez-Manzano, Arturo Ribagorda

**Affiliations:** Computer Security Lab (COSEC), Universidad Carlos III de Madrid, ES-28911 Madrid, Spain; lgmanzan@inf.uc3m.es (L.G.-M.); arturo@inf.uc3m.es (A.R.)

**Keywords:** user-in-a-context continuous authentication, smartphone data, data stream mining, non-assisted sensors

## Abstract

Smartphones are equipped with a set of sensors that describe the environment (e.g., GPS, noise, etc.) and their current status and usage (e.g., battery consumption, accelerometer readings, etc.). Several works have already addressed how to leverage such data for user-in-a-context continuous authentication, i.e., determining if the porting user is the authorized one and resides in his regular physical environment. This can be useful for an early reaction against robbery or impersonation. However, most previous works depend on assisted sensors, i.e., they rely upon immutable elements (e.g., cell towers, satellites, magnetism), thus being ineffective in their absence. Moreover, they focus on accuracy aspects, neglecting usability ones. For this purpose, in this paper, we explore the use of four non-assisted sensors, namely battery, transmitted data, ambient light and noise. Our approach leverages data stream mining techniques and offers a tunable security-usability trade-off. We assess the accuracy, immediacy, usability and readiness of the proposal. Results on 50 users over 24 months show that battery readings alone achieve 97.05% of accuracy and 81.35% for audio, light and battery all together. Moreover, when usability is at stake, robbery is detected in 100 s for the case of battery and in 250 s when audio, light and battery are applied. Remarkably, these figures are obtained with moderate training and storage needs, thus making the approach suitable for current devices.

## 1. Introduction

The use of smartphones has constantly raised in the last years. In 2017, the number of smartphones is estimated in 4.49 billions (i.e., 59.9 % of the population) worldwide [1]). Along with this generalization, security and privacy threats come into play. As an example, applications such as banking or shared document access require high levels of confidentiality. Authentication is one of the critical issues to achieve [2]. Thanks to authentication, it is possible to confirm that the alleged user carrying out an action is the one she claims to be. This issue is particularly relevant when it comes to protecting the usage of the smartphone itself. In this regard, a plethora of approaches have already been proposed, the use of Personal ID Numbers (PINs) or lock patterns being the most widespread ones [3].

One of the main drawbacks of the authentication mechanisms introduced so far is that authentication is typically performed once per usage session. Thus, if the device is robbed after carrying out a successful authentication, the attacker would gain all privileges associated with the authorized user. Therefore, it is essential to provide an advanced form of authentication that verifies the user presence at all times. This variant is called *continuous authentication* (CA) [4].

In order to continuously authenticate the user, several approaches have already been proposed. In particular, the use of soft biometrics (i.e., non-invasive biometric traces that can be obtained from the usage) has received extensive attention from the research community [5]. Apart from authenticating the user, another element that needs attention is her environment or context. Since users typically follow a set of habits in their daily routine, it is desirable to determine if these habits are kept in order to gain confidence on the usage normality. For example, if the user works in a library, it could be strange if a huge amount of noise is perceived. This observation could be taken into account to achieve progressive authentication—if the deviation from the routine is small or happens only once, perhaps no further action is required [6].

For the sake of security, both if the same user is in an unexpected environment, or if a different user is in the typical environment, may serve as an indicator of a potentially abnormal situation—robbery or use under coercion. In 2016, Basu et al. coined the term *user-in-a-context* for this research direction [7]. Indeed, since 2010, a significant amount of works (such as [8,9,10,11,12]) have already addressed it. However, most existing proposals make extensive use of assisted sensors, that is, sensorial information that is retrieved by cooperation with an external element (e.g., GPS satellites, cell tower or WiFi router) or magnitude (e.g., magnetism). Therefore, in case this element is unavailable, the detection mechanism is affected. To the best of authors’ knowledge, the only exception is the work by Lee and Lee [8]. Nevertheless, the significance of their work is limited due to the extremely reduced dataset (four users, five days). On the other hand, existing works usually focus on security issues, that is, achieving a high authentication accuracy. However, usability issues cannot be neglected—from the practical point of view, ensuring that the mechanism does not reject the legitimate user is also relevant. Furthermore, it would be desirable to make the system be tunable, prioritizing security or usability depending on user preferences.

To the best of authors’ knowledge, no previous work has addressed this issue.

To overcome these limitations, in this paper, a mechanism for joint detection of user and environment is proposed. The mechanism leverages existing data stream mining techniques, which are suitable to process continuous streams of data with moderate computational requirements [13]. These techniques may be the result of adapting previous data mining approaches, or entirely new methods suitable for data streams. Concerning the data at stake, four sensorial data streams (ambient noise and light, battery consumption and transmitted data) are considered, measuring their capacity (alone or in combination) to serve as identifiers for the user and her environment. These sensorial data streams do not rely upon any external element, thus increasing the detection autonomy. Moreover, the approach is evaluated considering both security and usability aspects using a dataset of 50 users during 24 months.

In short, the main contributions of this paper are:

- We explore the use of data stream mining techniques over smartphone sensor data to achieve user-in-a-context authentication.
- We study the extent to which four non-assisted sensorial data streams (battery, transmitted data, ambient noise and light) can serve as identifiers, either alone or in combination.
- We determine how long it takes to detect robbery leveraging the aforementioned data streams under different usability, immediacy and readiness constraints.

**Paper organization**. The remainder of this paper is organized as follows. Section 2 offers a background to the reader. Section 3 introduces the model and assumptions. Section 4 describes the proposed mechanism. Section 5 introduces the evaluation of the proposal, whereas Section 6 shows the related work. Finally, Section 7 concludes the paper and points out future work directions.

## 2. Background

This section introduces two issues that are relevant for the proposal. First, the concept of data stream mining is described. Afterwards, the sensorial information available in smartphones is introduced.

### 2.1. Data Stream Mining

Data stream mining techniques are a particular branch of machine learning procedures [13]. In machine learning, typically a pattern or feature is learned, which helps to classify a given set of elements into different classes. For this purpose, the dataset is usually divided into two subsets, namely training and testing. Whereas the first one is used to build the data model to make future predictions, the second one is applied to verify its effectiveness for classification.

Despite the great development of machine learning, this technique faces limitations in some settings [13]. In particular, it is not suitable for cases in which there is no pre-known, fixed-size dataset, but a potentially infinite one. On the other hand, it cannot be applied when the computational resources do not allow for storing all elements in memory.

To address these shortcomings, Data Stream Mining (DSM) techniques have been proposed [14]. DSM is suitable for settings in which data is received in a continuous stream. In such a case, it is unfeasible to keep storing values in order to get a dataset. On the contrary, DSM processes elements using a queue, where elements are processed only once. Then, DSM techniques are intended to learn incrementally based on the order in which data samples are received.

In particular, DSM is a suitable alternative when a continuous supply of data is at stake due to the following DSM established requirements [13]:

- *Process an example at a time and just once*: Data should be processed as it arrives. When an instance is discarded, there is not any possibility to retrieve it again. This limitation can be relaxed only in cases where it is practical to re-analyse an entire data stream. Since smartphone sensors produce endless data streams and count on constrained storage, for the purpose of this work, this requirement is critical.
- *Use of a limited amount of memory*: DSM techniques should be able to process much more data than memory available. Memory is commonly used to store running statistics and to store the current model.
  As in the previous case and given the limitations mobile devices have, this requirement is essential in the proposed context.
- *Work in a limited amount of time*: An algorithm must scale according to the number of instances, thus complexity should be linear in the number of samples. DSM algorithms should process data at real time, ensuring that data is processed according to the stream speed. Time is more or less critical depending on the application. Nonetheless, algorithms should be as fast as possible to give results in a sensible amount of time.
  In the considered case, the process should be carried out with the minimum possible delay to timely detect robbery.
- *Predict at any point*: the best algorithm should do the best prediction regardless of the amount of analysed samples. Generating a model should be as efficient as possible, avoiding recomputations in the final stage. Due to this issue, there is not a training phase if it is defined as the period needed to set up the system before entering in production.

For their interest for this work, we herein introduce use Naive Bayes (NB), K-Nearest Neighbor (KNN) and Hoeffding Adaptive Trees (HAT) algorithms. According to Bifet et al. [13], these are representatives of three of the main classes of DSM techniques. It must be noted that NB and KNN exist for traditional data mining (i.e., batch learning) settings, in which they cope with a previously known dataset of fixed size. However, we describe their adapted versions for DSM:

- Naive Bayes (NB). This technique naturally keeps a moderate memory usage over time. It consists of assigning the most probable class for an instance *X* based on the classes assigned to previous instances, using Bayes theorem. Let assume the set {*X*, *C*}, where *X* = {$x_{1}$, $x_{2}$, …, $x_{n}$} are the *n* most recent instances and *C* = {$c_{1}$, $c_{2}$, …, $c_{n}$} are classes of related users. It must be noted that, in the adaptation to DSM, this technique only keeps a subset of all already seen instances in memory. Given a new instance $x_{i}$, the use of NB involves computing [15]:
  (1) $$P\left(C=c_{j}|X=x_{i}\right)\propto P\left(C=c_{j}\right)\prod \left(P\left(X=x_{i}|C=c_{j}\right)\right).$$
  The predicted class is the one that maximizes $P(C=c_{j}|X=x_{i})$.
- K-Nearest Neighbor (KNN). It is a lazy algorithm, meaning that all the effort is performed once predictions are required. It looks for already known instances that are more similar to the instance to classify. In particular, the used algorithm classifies an instance based on its *k* nearest neighbors according to a distance metrics. In our case, the distance d(x,y) is computed using the Euclidean distance. For bi-dimensional points, this is computed as follows:
  (2) $$d\left(x,y\right)=\sqrt{{\left(x_{1}-x_{2}\right)}^{2}+{\left(y_{1}-y_{2}\right)}^{2}}.$$
  Any instance is classified considering the predominant label of the *k* nearest ones. It must be noted that, in the DSM version, these points are a subset of all instances seen at the moment in which the prediction is done.
- Hoeffding Adaptive Trees (HAT). In order to understand HAT, the concept of Hoeffding Window Tree (HWT) is required. A HWT is a decision tree based on a sliding window keeping the last instances on the stream. This type of trees use the Hoeffding bound [16], which states that, with probability 1-δ, the true mean of a random variable of range *R* will not differ from the estimated mean after *n* independent observations by more than:
  (3) $$\epsilon =\sqrt{\frac{R^{2}\cdot \left(ln\left(\frac{1}{\delta }\right)\right)}{2\cdot n}}.$$
  Based on this concept, a HAT is a HWT that learns from data streams without a fixed size of the sliding window. In this paper, we focus on the ADWIN variant, which automatically and continuously detects the rate of change in data streams rather than using a priori guesses [17].

### 2.2. Sensorial Data in Smartphones

Current smartphones are equipped with a significant set of sensors [18]. They can be divided into two main groups according to their autonomy, namely non-assisted sensors and assisted ones. The first group is formed by all sensors that can operate without any external element. Thus, readings such as the amount of data transmitted, the touch events or the external temperature belong to this group. One important advantage of these sensors is that they are fully autonomous if the device is operating in the allowed working conditions.

The second group of sensors is formed by assisted ones. This group contains all sensors that require some immutable element to operate correctly. Thus, GPS sensors belong to this group since they need to communicate to a subset of satellites (or base stations) that form this positioning system. Similarly, accelerometer sensors rely upon reliable measurements of gravity. In terms of autonomy and proper operation, they are subject to the status of the external element.

## 3. Model

In this section, the underlying model of the considered approach is presented. Thus, the entities at stake and the capabilities of the adversary are introduced in Section 3.1. Afterwards, the goals to be achieved are described in Section 3.2. Finally, working assumptions are stated in Section 3.3. The notation used throughout the paper is shown in Table 1.

### 3.1. Entities: Adversarial Model

There are four entities in the considered model. On the one hand, the smartphone is possessed by an authorized user U. This entity is the typical holder of the device, and thus makes regular use of it. On the other hand, the second entity is the environment $E_{U}$ in which U makes use of the device. E may be formed not only by one physical location (e.g., U’s home or work) but indeed from different places according to the user habits.

As opposed to these two entities, both the adversary A and her typical environment $E_{A}$ come into play. The adversary is willing to rob the smartphone, bring it to $E_{A}$ and execute some applications or retrieve some data from the device. This action takes a time $T_{\mathrm{ROB}}$ to complete. In this way, the identification of the theft, $T_{\mathrm{SEC}}$, should be performed in the minimum possible time, such that $T_{\mathrm{SEC}}\leq T_{\mathrm{ROB}}$.

### 3.2. Goals

The proposed mechanism must meet the following goals:

- **Accuracy**. The smartphone should be able to differentiate between U and A (user identifiability), between $E_{U}$ and $E_{A}$ (environment identifiability) and between U in $E_{U}$ and A in $E_{A}$ (user-in-a-context identifiability).
- **Immediacy**. The smartphone should be able to detect the presence of A in $E_{U}$ or the robbery from $E_{U}$ to $E_{A}$ in a period of time $T_{\mathrm{SEC}}$ that should be as short as possible.
- **Usability**. The smartphone should only get blocked when A comes into play. The device should remain in safe mode (i.e., unblocked) while the user U is porting it in $E_{U}$. In addition, the device should store a small set of data to keep on working, thus relieving the storage capacity.
- **Readiness**. The mechanism should be able to start working in the smartphone after a learning period that should be as small as possible.

### 3.3. Working Assumptions

The mechanism is intended to work under the following conditions. First, the adversary A carries out a set of activities that are different from those of U. For example, the set of applications that A uses, or the way in which she does it, are different to some extent. In a similar way, $E_{A}$ should be different from $E_{U}$. Both conditions impose that mimicry attacks [19] in which the attacker tries to mimic the legitimate user habits, are left out of the scope. Similarly, we assume that the attacker will rob the device for a sensible amount of time. Thus, we leave the detection of micro-robbery (i.e., stealing the device for a few seconds) for future work.

## 4. Approach

This section introduces the proposed approach. For this purpose, Section 4.1 describes the authentication method. Afterwards, Section 4.2 describes the particular data items at stake.

### 4.1. Approach Description

The proposed continuous authentication method is intended to be carried out by the smartphone. The availability of real-time sensorial information enables the smartphone to analyze the current status of the environment, its actual computational status and the usage patterns.

**Figure 1:**
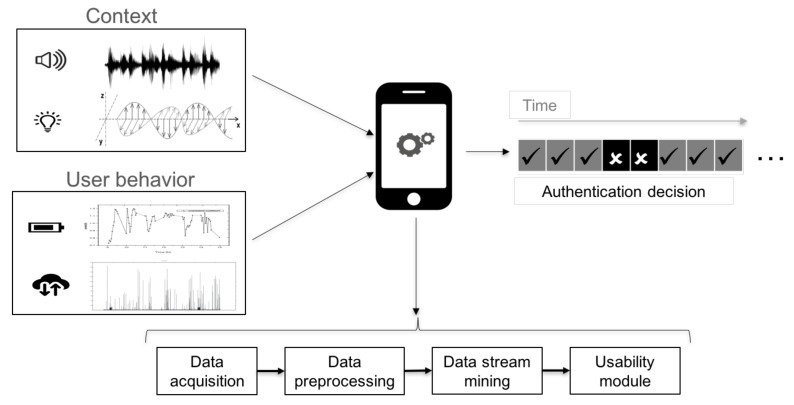
Overview of the proposed continuous authentication method.

Leveraging both sensorial data inputs and computational capabilities of smartphones, the proposed method works as follows (Figure 1). The first step is to retrieve the sensorial information, which is relevant to the mechanism. The actual set of sources that are considered for the proposed method are discussed in Section 4.2. With these data at hand, the second step is to prepare them to be further processed. This task involves discretizing and transforming data in some computationally-friendly representation.

Once this data is ready to be processed, the last step is to analyse it. In this regard, we opt for Data Stream Mining (DSM) techniques, which are suitable to make predictions on data requiring an affordable amount of resources (recall Section 2.1). Particularly, we consider three representative algorithms—Naive Bayes (NB), K-Nearest Neighbor (KNN) and Hoeffding Adaptive Tree (HAT). NB is chosen as its application to data streams is straightforward due to the small amount of required parameters to establish [20]. Moreover, its complexity is low since it only depends on computing a probability over each variable. On the other hand, KNN is selected as it is a popular research topic for DSM and has been successfully applied in prior works. Furthermore, it is a non-parametric lazy algorithm, which means that no assumptions over the data distribution are made. Last but not least, HAT is chosen because it is the current state-of-the-art in classifying high speed streams [13]. They naturally cope with streams that evolve over time, which could be the case taking into account the time span of the dataset (see Section 4.2).

Each of these methods outputs an authentication decision after each input data sample. Such a decision reflects whether the smartphone believes that it is in a known environment with the authorized user (that is, U and $E_{U}$) or not (that is, A or $E_{A}$). In the latter case, the smartphone can turn on some active authentication mechanism (e.g., request for PIN) or any other defensive measure. To achieve a tunable security-usability trade-off, we introduce a usability module, controlled by a parameter called sensitivity threshold ST. This value represents the actual number of consecutive readings through which the device has to detect the attacker presence in order to trigger the said defensive action. Intuitively, the smaller this value is, the more prone to errors is the mechanism. This undesired situation could lead to undue rejections of the legitimate user, thus having a negative impact on usability.

### 4.2. Sensorial Information

In order to identify U and $E_{U}$, it is necessary to retrieve some information. In particular, this mechanism leverages non-assisted sensorial data (recall Section 2). The user U is described by the way in which the battery is used and the amount of transmitted information (recall Figure 1). Both data sources are interesting since they are tightly coupled with the use that U makes of the smartphone. Thus, when U charges her device, or the speed in which it is discharged, may reveal a particular behavioral pattern from the user. Similarly, the amount of transmitted data is intrinsically linked to the usage of applications [21].

With respect to $E_{U}$, this mechanism relies upon the audio and light measurements of the environment. Both physical variables are interesting since they can be retrieved continuously and can be analyzed in both the time and frequency domains. Therefore, the amount of information they can offer may be especially valuable.

User data has been taken from the Sherlock dataset [22]. This dataset is formed by data from 50 users throughout 24 months. Table 2 summarizes the dataset considered for this paper.

For each of these sensorial sources, several data items are at stake (see Table 3). In total, 20 data items are considered for the ambient audio data stream, including band-power and frequency related descriptors (e.g., power spectral density [23], PSD, or Mel Frequency Cepstral Coefficients [24], MFCCS). With respect to ambient light, only the actual level of light in lumens is gathered, along with the accuracy measurement. Concerning battery, a total of nine data items are considered, including its temperature, its current charge and its status (e.g., plugged or not). Finally, for data transmission, both sent and received packets and bytes are considered in both mobile and Wi-Fi technologies.

## 5. Experimental Analysis

This section describes the experimental assessment carried out over the considered sensorial data. For this purpose, the analysis on accuracy is presented in Section 5.1, whereas Section 5.2 focuses on immediacy, usability and readiness issues. After the individual analysis of all features, Section 5.3 discusses what are the best settings to achieve a security-usability trade-off.

For the sake of repeatability, an Intel i7 computer with 4 GB. RAM has been used for data preparation. Although a Sherlock dataset is considered, further data preparation is needed since each of the studied features require a set of experimental files. On the other hand, an Intel i5 with 8 GB. RAM has been applied to carry out the data stream mining procedure, using Massive Online Analysis (MOA) tool in its version as of August, 2017 [25]. The experimental results are then processed with MATLAB R2016b in order to obtain $T_{\mathrm{SEC}}$ for each system setting. All the scripts developed for preparing the data, running the experiments in MOA and processing the results are publicly available in a GitHub repository [26].

### 5.1. Accuracy Analysis

This section studies if the considered data is useful for continuous authentication. For this purpose, the data stream mining algorithms introduced in Section 4 have been applied over each data source, as well as over combinations of them. Essentially, if a given data source is unique per element, it would be useful as an identifier. Therefore, this analysis is focused on determining whether audio and light measurements are identifiers for the environment and battery and transmitted data can identify the user.

In order to avoid any bias, this analysis is carried out in an all-vs.-all fashion, i.e., all sensorial data of all users are interleaved. In this way, the relevance of the results is ensured since it leads to having a global accuracy measurement. For the sake of clarity, accuracy is computed as the ratio of successful user authentications and the total amount of evaluations. More precisely, let TR be the total number of records for each particular combination of data sources. Recall that this set contains data for all ns subjects. Each record will be analyzed by the DSM technique at stake, leading to an authentication decision. Thus, at the end of the experiment, each user U has a number of correct authentications (succ${}_{U}$). The accuracy accy is then computed as a fraction between this amount for all ns users and TR, as shown in Equation (4):

(4) $$accy=\frac{\sum_{U=1}^{ns}succ_{U}}{TR}.$$

#### 5.1.1. Data Preparation

In order to carry out this analysis, all records for each type of data are gathered in a single file (e.g., audio, light, battery and transmitted data). These files are intended to perform an independent analysis on each feature.

**Figure 2:**
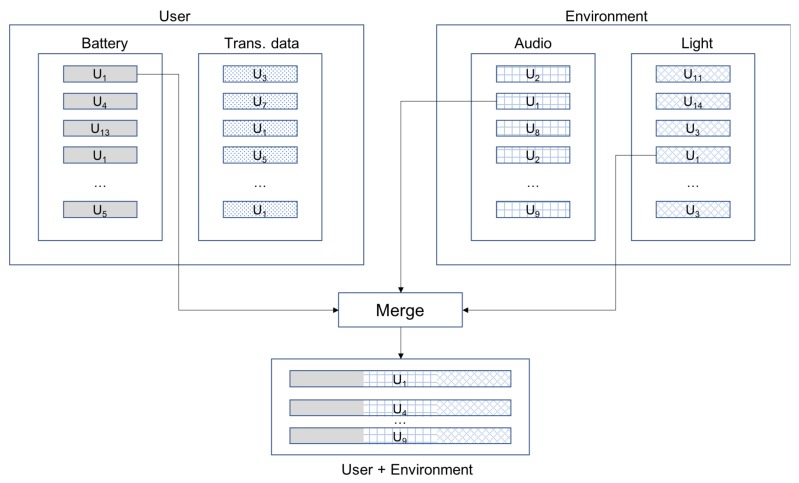
Data preparation for accuracy analysis (Audio + Light + Battery case).

On the other hand, different features are combined as well. In this way, it is possible to determine whether the addition of features improves the identification capability. Moreover, it also enables achieving the goal of identifying a user-in-a-context. For this purpose, it is necessary to build records that gather information from both the user and the environment. This is done by merging the independent files described in the previous paragraph. For illustration purposes, Figure 2 describes how the input data file is built for the combination of audio, light and battery sensors. Since environmental variables are sampled every 10 s, whereas user-related ones are sampled every 5 s (recall Table 2), it is necessary to adjust both information sources. In particular, the merged records are built by taking every environment-related records and one out of two user-related ones.

#### 5.1.2. Analysis Results

The result of this analysis is summarized in Table 4. Concerning the environment, the best results are achieved when both audio and light are considered together (68.29%). It must be noted that light alone offers a very limited identifying capability (43.31%). Although further research would be needed, it may be due to the fact that, when the device is carried in a bag or in a pocket, the light values are more affected than noise. Thus, some noise can be perceived by the device even if it is held in a bag.

Concerning the user, battery readings offer a high capability as identifier (97.05%). On the contrary, transmitted data is very weak as identifier (18.51%). One of the main reasons is that mobile devices are continuously exchanging information with other entities, thus reducing the value space.

Considering both the user and environment together (i.e., user-in-a-context identifiability), the combination of audio, light and battery offers a remarkable accuracy (81.35%). Intuitively, it is reasonable that the achieved value is between the user and environment identification accuracies, since the data at stake is formed by both elements together.

These findings point out to what extent these sensorial data sources are unique and distinguishable at a given point in time. In other words, the system is able to learn the values expected for each user and classify them accordingly. One important matter is that this decision is not only based on each reading—previous values are taken into consideration. In other words, the order in which data is received is meaningful by itself. To confirm this belief, another side experiment was carried out. Considering the data stream with highest accuracy (i.e., battery), a new data stream was built by randomly shuffling the readings. After this operation, the method achieving the best results (i.e., KNN) was applied, leading to an accuracy of 7.34%. In light of this value, it is confirmed that the order of the readings has a direct impact on accuracy.

### 5.2. Immediacy, Usability and Readiness Assessment

The previous accuracy results are encouraging since they suggest that these data are useful to tell users (and their environments) apart. However, the actual case in which this issue is relevant is when the device is robbed. In this case, the three remaining goals come into play. Thus, it is necessary to detect this fact as soon as possible (immediacy), avoiding the rejection of the actual user (usability) and requiring a short amount of time for learning from the user to work properly (readiness).

This section starts by the experimental preparation (Section 5.2.1), which describes the data and parameters at stake. Afterwards, the actual assessment of immediacy (Section 5.2.2), usability (Section 5.2.3) and readiness (Section 5.2.4) is presented.

This assessment is based on determining how $T_{\mathrm{SEC}}$ is affected by the parameters that are closely related to each goal. Therefore, the experiments for each goal consist of fixing values for its related parameters and considering all possible values for the remaining parameters. Hence, plots show the distribution of $T_{\mathrm{SEC}}$ for all these experiments. We opt for $T_{\mathrm{SEC}}$ since it serves as an indicator of the system effectiveness and has a direct real-world meaning. On the other hand, since the proposal looks for achieving a security-usability trade-off, all figures also show the impact of the usability parameter ST. For the sake of clarity, the boxplot representation is adopted. Boxplots, proposed by Tukey in 1977, are relevant to show the distribution of data in a compact way [27]. Thus, the edges of the box show the first and third quartiles (i.e., 25% and 75% of values), whereas the red line shows the median. Moreover, whiskers extend to those points that are not considered *outliers*. In our experiments, outliers are those values beyond 1.5 times the Inter-Quartile Range (IQR), that is, the difference between the aforementioned quartiles. Thus, whiskers extend up to those values until 1.5 IQR times the 25% quartile, and those until 1.5 IQR times bigger than the 75% quartile. This is a typical decision for boxplots, being the default configuration of Matlab ones [28].

#### 5.2.1. Experimental Preparation: Data and Parameters

Based on the files prepared for the previous experiment (recall Section 5.1.1), a new set of files are created for this analysis. In particular, it is needed to have the device stolen by A and measure $T_{\mathrm{SEC}}$, that is, how fast it is possible to detect this issue.

We study user-vs.-attacker situations in order to achieve this goal, thus analysing when A is in $E_{U}$ or that a robbery takes place from $E_{U}$ to $E_{A}$. Thus, robbery is simulated by creating files with data from the legitimate user U followed by data from any other user in the dataset, which acts as an attacker A. In particular, these files are created assuming that A takes the device at a particular time *t* and from that moment on the device is considered stolen and in the hands of the attacker. To avoid any bias, a pair of issues are carried out: (1) these experiments involve 10 randomly chosen subjects acting as U, whereas other 10 randomly chosen subjects act as A; and (2) between readings of U and A a set of instances have been introduced to simulate the robbing action. Thus, there is a smooth transition between both user readings. Otherwise, the experiments would not be realistic since, for example, the device could move from a fully quiet environment to a crowded and noisy one in just a single reading (i.e., 5 s). In our experiments, we have introduced a set of 10 records for this purpose.

The design of these files has a direct impact into the type of analysis that can be carried out. Given that the DSM technique at stake will learn from U, and afterwards will receive readings from A, a typical accuracy analysis is not informative. Indeed, no false positives may occur, as a consequence of this design. However, false negatives will happen—this is what $T_{\mathrm{SEC}}$ actually measures. Therefore, this magnitude is a real-world illustration of false negatives occurring until the system determines the attacker presence.

In order to carry out this analysis, different combinations of sensorial features have been taken into account. In particular, for this analysis, the three best identifiers (recall Section 5.1.1) have been considered: the combination of audio and light (AL) for identifying the environment, the battery (B) to identify the user and the combination of these three (ALB) to identify a user-in-a-context.

Apart from the sensorial features, different periods for learning from U are considered. In particular, 500, 2000, 5000, 8000 and 10,000 readings are chosen. In this way, it is possible to determine whether the amount of available knowledge about U leads to lower $T_{\mathrm{SEC}}$ rates. On the other hand, the robbery period ($T_{\mathrm{ROB}}$) has been set to 5000 readings. As long as this period is equivalent to around 7 h (in the case of battery), we believe that it should be enough to detect any robbery.

Considering all these variables (i.e., amount of users, sensorial data and learning periods), the amount of files involved for these experiments is calculated as follows:

10(users)×3(sensorialdata)×5(learningperiods)=150.

With respect to experimental parameters, KNN is the only algorithm considered as it is the one that achieved the best accuracy results (recall Section 5.1). In terms of parameter *k* of this algorithm, values 3, 10 and 21 are established. On the other hand, instances stored in the device may be 1000, 5000 and 10,000. Concerning the value ST (recall Section 4.1), we take the values ST={1,10,20}, recalling that ST=1 means 5 s for B and 10 s for AL and ALB.

Apart from these general parameters, immediacy assessment requires an additional setting. In particular, it is interesting to compare the effectiveness of the system in the user-vs.-attacker situation (i.e., the one described so far) with an artificial user-vs.-user one, which is U in $E_{U}$ at different times pretending to be A. In this artificial user-vs.-user case, we make the same user be the attacker by including some of her readings pertaining to another moment in time. This setting (which does not have any real meaning) is useful to confirm the hypothesis that it should be easier to detect a real attacker sooner, as a natural consequence of the accuracy results. In other words, it is expected that a user is more similar to himself than to anyone.

#### 5.2.2. Immediacy Assessment

There are three factors that can have an impact on the immediacy, namely the difference between user-vs.-attacker and user-vs.-user settings, the sensor(s) at stake and the choice of *k*. Each issue is analysed in the following.

**Figure 3:**
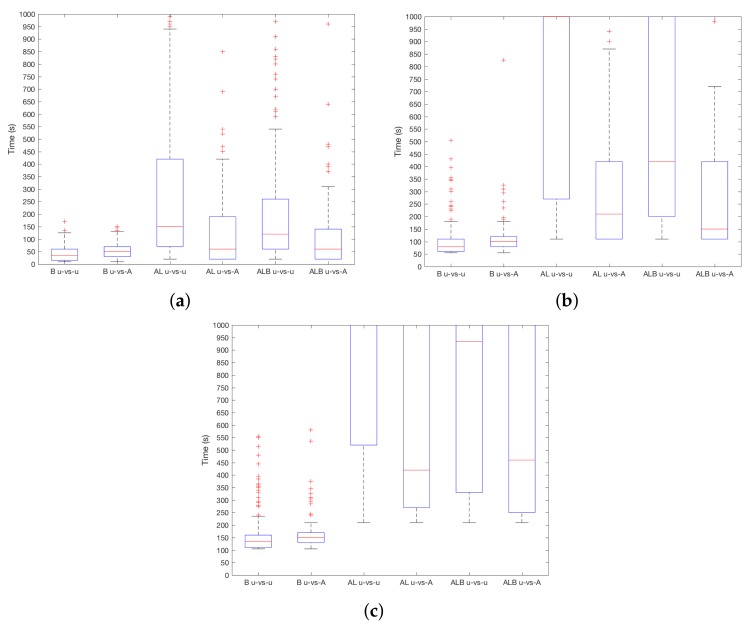
$T_{\mathrm{SEC}}$ results for user-vs.-attacker and user-vs.-user settings. (**a**) ST=1; (**b**) ST=10; (**c**) ST=20.

The results of $T_{\mathrm{SEC}}$ in the user-vs.-attacker and user-vs.-user situations are shown in Figure 3a–c. As it can be seen, results for AL and ALB confirm expectations—regardless of the chosen ST, the system detects robbery easier when the robber is A instead of U, thus in a user-vs.-attacker setting. This difference is more important for higher ST. For instance, for AL and ST=10, the median decreases from 1000 s in a user-vs.-user situation, to 200 s in a user-vs.-attacker situation. For ALB and ST=10, detection is performed in 450 s and 950 s in user-vs.-attacker and user-vs.-user situations, respectively. On the contrary, in the case of B, the difference between both situations is almost equivalent. For instance, for ST=20, detections are carried out at 141 s and 150 s for user-vs.-user and user-vs.-attacker situation. In practice, it means that the system is as good distinguishing between A in $E_{U}$ as between A in $E_{A}$ at different times in the case of B, while, for AL and ALB, the system is better at spotting the attacker, i.e., distinguishing between A in $E_{U}$.

**Figure 4:**
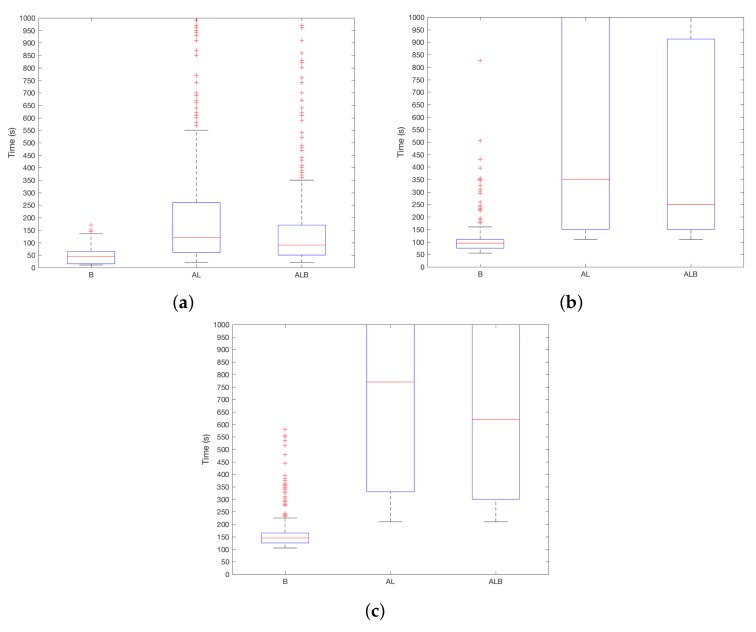
$T_{\mathrm{SEC}}$ results depending on the chosen sensors. (**a**) ST=1; (**b**) ST=10; (**c**) ST=20.

With respect to the type of sensor at stake, Figure 4a–c shows the results considering different ST values. In general, B works better and robberies are detected faster. When ST=1, the median is around $T_{\mathrm{SEC}}=50\;$ s, while AL and ALB are close to $T_{\mathrm{SEC}}=100$ s. With the increase of ST, the difference increases, and ALB outperforms AL. This means that B adds useful information to discriminate between users. Then, when ST=10, using B, the device will detect the attacker after $T_{\mathrm{SEC}}=100$ s on median, $T_{\mathrm{SEC}}=250$ s in the case of ALB and after $T_{\mathrm{SEC}}=350$ s using AL. This difference is higher for ST=20–using B robbery is detected in $T_{\mathrm{SEC}}=150$ s, and $T_{\mathrm{SEC}}$ = 750 s and $T_{\mathrm{SEC}}=600$ s for AL and ALB, respectively. In addition, it must be noted that quartile 3 (i.e., 75% boundary) for AL and ALB is beyond $T_{\mathrm{SEC}}=1000$ s. Therefore, the use of the device (i.e., information from B sensor) is more decisive than the environmental conditions (i.e., information from AL sensors) to distinguish between users.

**Figure 5:**
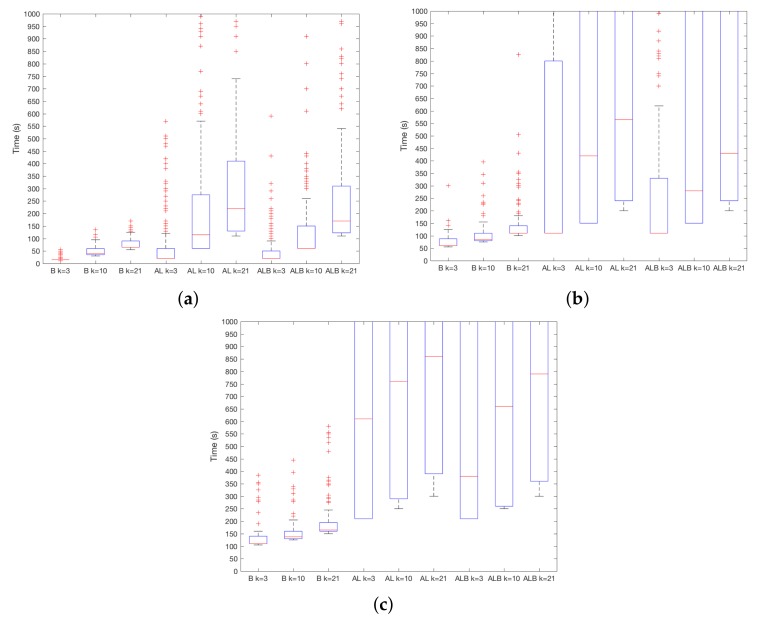
$T_{\mathrm{SEC}}$ results depending on parameter *k*. (**a**) ST=1; (**b**) ST=10; (**c**) ST=20.

Last but not least, the impact of *k* for $T_{\mathrm{SEC}}$ is shown in Figure 5a–c. As it can be seen, smaller values work better independently of the sensor and ST. Intuitively, this can be a direct consequence of the inner workings of KNN—greater values of *k* lead to a higher probability of having readings from U as part of such *k* points. If a majority is reached when values from A are evaluated, the incorrect category is chosen. Differences are higher when ST increases, for instance, for AL when ST=10, the difference between medians of every *k* is of 300 s.

#### 5.2.3. Usability Assessment

There are two issues that have an impact on usability. On the one hand, the value of ST itself, which determines the hardness to apply the protective measures such as self-blockage. On the other hand, the amount of storage required for the mechanism to work properly. The first parameter may cause the system to sooner detect the attacker at the expense of improper blockages when the legitimate user is at stake. The second one determines whether this mechanism can be applied in regular devices or requires greater storage capabilities. Both issues are studied below.

In order to analyse the effect of ST, previous Figure 3, Figure 4 and Figure 5 have to be revisited. ST=1 means that the first time the system concludes that the user is A, it will activate its protection. In practice, the best achievable $T_{\mathrm{SEC}}$ would be 5 s for B and 10 s for AL and ALB, respectively. As it can be seen in the said figures, this optimal value is never achieved. Increasing ST to 20, the minimum detection time is $T_{\mathrm{SEC}}=20\times 5=100\;s$ for B and $T_{\mathrm{SEC}}=20\times 10=200\;s$ for AL and ALB. In practice, $T_{\mathrm{SEC}}$ is far beyond that value. However, it is expected that this would make improper self-blockages to be less likely—now, 20 consecutive errors (i.e., U or $E_{U}$ being recognized as A or $E_{A}$, depending on the type of sensor) should happen.

**Figure 6:**
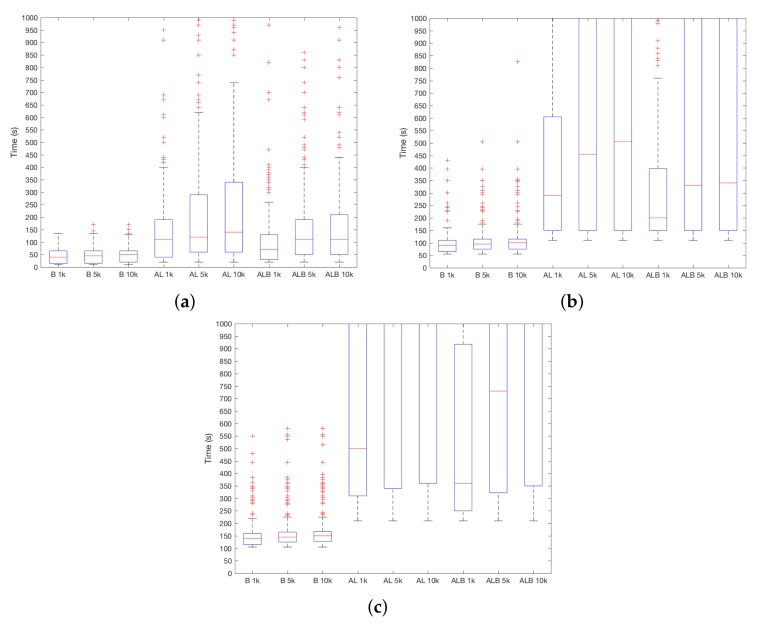
$T_{\mathrm{SEC}}$ results depending on the amount of storage required. (**a**) ST=1; (**b**) ST=10; (**c**) ST=20.

Regarding the effect of the required storage, it must be noted that it is preferable to request a minimal amount of storage to ensure the suitability of the approach to regular user devices. Before entering into the experimental results, it is paramount to take into account some relevant details of the DSM technique at stake. In this adapted version of KNN, only a subset of readings are kept in memory. Thus, a sliding window is applied to limit the storage space. This window works in a first-in, first-out policy—the oldest reading is discarded to make room for the freshest one. Therefore, the storage size can be regarded as a synonym for the window size. On the other hand, KNN considers the *k* nearest neighbors in order to assign the label for the reading at stake (recall Section 2). Therefore, the probability of assigning a label of one class (i.e., user or attacker) grows with the presence of readings of that class. With these issues in mind, let us consider Figure 6a–c in which this matter is analysed. As expected, the bigger the storage size (i.e., the window size), the longer it takes for the system to determine the attacker presence. Indeed, with an storage of 1000 readings (i.e., around 1:30 h. of battery consumption), promising detection rates can be achieved.

Measuring the size in terms of readings offers little information in what comes to determine the suitability of this approach to current devices. To study this aspect, Table 5 presents the amount of bytes that have to be stored per reading for each particular sensor. Values have been established based on the worst case considering all managed data and *separators* (e.g., comma) correspond to elements used to separate every stored value per reading. For instance, given the storage of 1000 readings, in the case of batteries, 1000 × 26 = 2600 B (2.6 Kb) have to be stored. Another example is ALB, in this case, 1000 × (26 + 6 + 380) = 380,032 B (380 Kb) should be stored. As the average size of photos in a smartphone such as the iPhone6S is 2.59 Mb [29], the size of sensor readings to be stored in the device is complete sensible, i.e., much smaller than the size of a picture. According to these results, storage requirements are affordable for current smartphone devices.

#### 5.2.4. Readiness Assessment

In order to assess readiness, it is necessary to measure the amount of training data that is required for the mechanism to work, that is the length of the learning process. In other words, this analysis focuses on measuring the extent to which the proposed mechanism benefits from the fact that the authorized user is porting the device for a period of time before the robbery.

To understand the results, it is essential to recall that DSM techniques limit the amount of used memory. This means that only a subset of the freshest readings are considered when analysing a new input. Moreover, KNN works by labeling the input with the label of a majority of the *k* nearest neighbours. Thus, in practice, no knowledge from the readings removed from memory is considered when analysing a new input. In other words, the system does not build a monotonic incremental model of the user.

**Figure 7:**
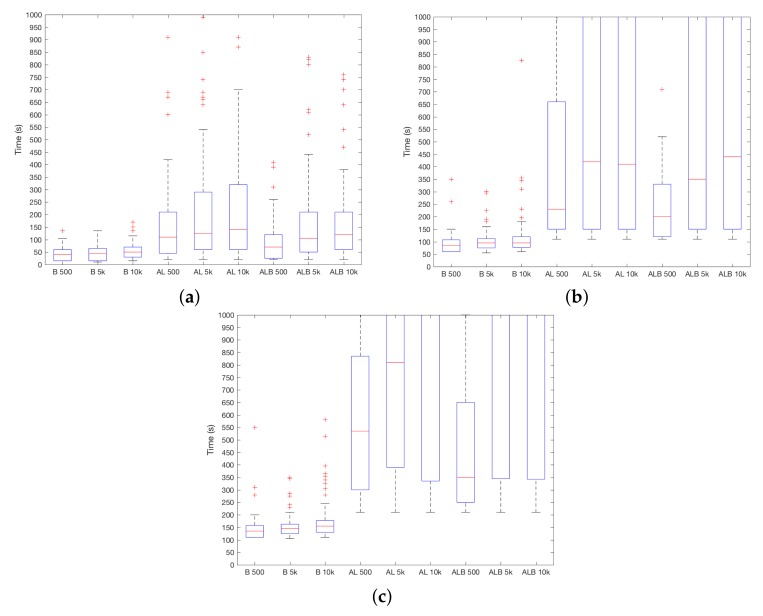
$T_{\mathrm{SEC}}$ results depending on the length of the learning period. (**a**) ST=1; (**b**) ST=10; (**c**) ST=20.

Considering these aspects and in line with expectations, Figure 7a–c show that $T_{\mathrm{SEC}}$ is promising after a short user learning process. The best choice is the use of B sensor, since the detection time is bigger with the remaining sensorial sources. However, it must be recalled that B is sampled twice as fast as both A and L sensors. Going into the details, the detection time remains below two minutes when the system learns from the user for around 6.9 h (i.e., 5000 readings) using B sensor and a moderate level of usability (i.e., ST=10). Interestingly, with that usability level as a limit, it can be seen that an increase on the size of the learning process (particularly when moving from 5000 to 10,000 readings) does not lead to a valuable benefit.

In light of these results, it becomes clear that KNN imposes an effective threshold on the amount of knowledge the system gets from the user. Indeed, it is limited by the amount of instances that are kept in memory.

### 5.3. Discussion: Towards the Best Settings for a Security—Usability Balance

The previous analyses have shown how the different parameters and settings affect each of the goals in the proposal. However, it is necessary to have a global view of which configuration achieves a nice trade-off between security and usability.

According to the obtained results, the use of battery readings with KNN and k=3 achieves the best results. In particular, after 500 readings for learning from the user (which is equivalent to 2500 s) and requiring the storage of 1000 readings, the mechanism is ready to work properly. In order to keep a balance between security and usability, ST=20 seems to be the best choice to promote fast detection while reducing the likelihood of improper protection. In this way, robbery can be detected in 150 s for ST=20, while in 100 s for ST=10.

In addition, though AL and ALB are not the best alternative, they get their best results with the same setting as the battery, that is with KNN and K = 3, as well as for 1000 readings. However, ST should be limited to 10 because $T_{\mathrm{SEC}}$ is significantly high in more challenging cases. In particular, for ALB (which get better results than AL), robbery can be detected in 600 s when ST=20 and in 250 s when ST=10.

## 6. Related Work

Since 2010, a plethora of contributions have worked on Continuous Authentication (CA) in smartphones. Table 6 summarizes the main existing works up to date. Note that the presented approach is included in the last row of this table to clearly identify differences between existing works and the proposed one.

Studied works (28 in total) usually consider a different set of sensorial features, such as location data (e.g., GPS traces), system-level information (e.g., CPU or battery) or input–output data (e.g., camera or microphone). In this regard, our proposal is in line with previous works as it involves a multi-sensor CA system. In the remainder of this analysis, we will only focus on systems in which smartphone sensors are at stake, thus leaving other works (e.g., [30], based on biosignals) out of the scope.

With respect to the purpose of the CA system, there is no precedent working on the authentication of the context alone. Indeed, most existing works (19 out of 28) focus on authenticating the user, with no attention to his/her environment. The remaining nine papers focus on the user-in-a-context authentication, thus addressing the same issue as our manuscript. For the sake of brevity, only the differences with these approaches will be highlighted.

In terms of the sensorial data at stake, it is remarkable that almost all precedent works [9,10,11,12,31,32,33,34,35] rely upon some external element such as GPS satellites or telephony cell towers. This issue is relevant as the absence of these elements would make their approach to be affected. Since this mechanism could be useful to detect robbery, autonomy of the device is critical to ensure its effectiveness. Therefore, as compared to previous works, our approach increases the smartphone autonomy as it does not require any external element. The only exception is the work presented in [8]. However, their dataset (four users in five days) is significantly smaller than the one considered in this work, thus making comparisons is very hard for the sake of fairness.

Concerning the experimental settings and results, our work does not achieve the best accuracy results. However, it must be noted that our work is the only one that takes both security and usability into account. Remarkably, none of the preceding works propose a tunable approach that balances security and privacy. In our approach, the use of ST enables prioritizing usability at the expenses of losing immediacy or accuracy. Our approach also achieves promising immediacy results as compared to previous works. Moreover, readiness has been studied in terms of the period of time for learning from the user. Although DSM does not take a training period before entering into production (as it has to be ready to predict at any time), for the sake of comparison, we confront our approach against the training needs of previous works. Most approaches establish a particular configuration, i.e., 80% of data for training [36], and just six of them try assorted values. In a different way, because our approach does not directly use training as it is generally understood, we consider that a mobile device can be robbed under five different settings, which is related to the time the system learns from the user until the robbery happens.

From the industry side, Google has already proposed a mechanism called Smart Lock for Android. According to the website [37], Smart Lock is intended to lock the device when it is not ported by the user. To do this, Smart Lock may leverage Bluetooth or Near Field communications (NFC) connections, trusted (i.e., pre-defined) locations or based on different biometric traces. In particular, Smart Lock may detect if a user is carrying the device, or may apply voice or facial recognition. Smart Lock already points out some limitations. Regarding trusted places [38], *trusted location can extend beyond the physical walls of your home or custom place. It can keep your device unlocked within a radius of up to 80 m*. Moreover, Google also states that *location signals can be copied or manipulated. Someone with access to specialized equipment could unlock your device*. Concerning on-body detection, Google states that it is less secure than other typical mechanisms such as PIN. As a result, in comparison with Smart Lock, our mechanism (1) does not leverage a predefined set of trusted places, thus adding flexibility to real-life user habits and (2) does not consider biometric traces, which could be subject to forgery.

## 7. Conclusions

Existing smartphone devices are provided with a significant set of sensorial information. These sensors may be applied for different purposes. In this work, their application to continuous authentication has been explored. Particularly, a subset of sensors has been considered. Interestingly, these sensors do not rely upon any external element, thus promoting the device autonomy to take authentication-related decisions. Experimental results using data stream mining techniques have shown that by combining battery consumption, ambient audio and light readings, it is possible to achieve good accuracy and immediacy. Remarkably, these goals are achieved while requiring a limited amount of time for learning from the user and taking into consideration usability issues.

As future work, mimicry attacks could be considered. For this purpose, advanced sensorial data combination techniques could be applied, including a richer set of sensorial information. Moreover, we will assess the impact of the amount of transition records that are introduced to simulate the robbery. The relationship between accuracy and the amount of data, especially in terms of randomization and battery drain, is another issue to be addressed.

## Figures and Tables

**Table 1:** Notation.

Symbol	Meaning
U	Legitimate user of the device
A	Attacker
$E_{U}$	User physical environment
$E_{A}$	Attacker physical environment
$T_{\mathrm{ROB}}$	Time needed by A to attack
$T_{\mathrm{SEC}}$	Time to detect robbery
A	Ambient noise sensor
L	Ambient light sensor
B	Battery consumption sensor
TD	Transmitted data sensor
ST	Sensitivity threshold

**Table 2:** Dataset description.

Data Item	Sampling Freq.	Population Size	Time Frame	Total Records
Ambient audio	10 s	50 users	24 months	98,850,425
Ambient light	10 s			76,072,797
Battery	5 s			203,472,430
Transmitted data rates	5 s			203,260,663

**Table 3:** Data items per sensorial data source.

Sensorial Source	Data Items
**Audio**	DiffSecs, PSD (4), MFCCS (12), Mathematical norms (3)
**Light**	Accuracy, Lux
**Battery**	Charge type, health, level, online, plugged, scale, status, temperature, voltage
**Transmitted data**	Mobile Tx/Rx packets (2), Mobile Tx/Rx bytes (2), WiFi Tx/Rx packets (2), Wifi Tx/Rx bytes (2), Total Tx/Rx packets (2), Total Tx/Rx bytes (2)

**Table 4:** Accuracy analysis.

	Source	KNN	Adaptive Hoeffding Tree	Naive Bayes
**Environment**	Audio (A)	63.70%	39.46%	7.58%
	Light (L)	43.31%	44.32%	8.61%
	A + L	68.29%	42.83%	7.58%
**User**	Battery (B)	97.05%	37.04%	4.78%
	Transmitted data (TD)	18.51%	10.33%	1.06%
**User + Environment**	A + L + B	81.35%	43.02%	12.02%
	A + L + TD	65.80%	24.64%	8.31%

**Table 5:** Bytes stored per reading for each type of sensor.

Sensor	B	Bytes	L	Bytes	A	Bytes
	Charge type	1	accuracy	1	DiffSecs	18
	Health	1	lux	4	PSD	47
	Level	3	*Separators*	1	MFCCS	72
	Online	1			Math. Norms	224
	Plugged	1			*Separators*	19
	Scale	3				
	Status	1				
	Temperature	3				
	Voltage	4				
	*Separators*	8				
**Total bytes**		26		6		380

**Table 6:** Related work comparison.

	Purpose			Sensors						Dataset size		Results			
Paper	Context CA	User CA	User- In -Context CA	App Usage Sensors (CPU, Priority, Use of Memory, Transmitted Data)	System-Related Sensors (CPU Usage, Memory Usage, Network Usage, Battery Usage)	Location- and Movement Sensors (Accelerometer, Rotation, GPS, Barometer, Cell ID)	Data-Related Sensors (WiFi, Bluetooth, Phone Calls, SMSs, Global Rx/Tx data)	IO Sensors (Audio, Light, Camera, Screen Info, Temperature)	Other Features	Amount of Users	Time Frame (Days)	Accuracy	Immediacy	Usability	Readiness (Training Conf. (%))
[22]		×		App CPU, memory, transmitted data	Battery, CPU, memory		WiFi, global Rx/Tx data			50	547.5	10${}^{-5}$ FRR	0.25 min/6.6 min	N/A	1
[36]		×				Accelerometer, gyroscope and magnetometer				20		71.3%	N/A	13.1% false alarms, period unknown	1 (80%)
[39]		(activity recogn.)			Battery	Accelerometer, magnetometer, GPS, rotation matrix	WiFi, global Rx/Tx data, bluetooth	Ambient light, audio, temperature, camera, microphone	Process list	310	150	57%	N/A	N/A (in the terms of this paper)	1
[8]			×			Orientation, magnetometer, accelerometer				7/25/100	21/365/183	90.23%	20 s	N/A	1
[9]			×	App	Battery, CPU	Orientation, magnetometer, accelerometer, rotation	WiFi	Light	Device active, call history	7/25/100	21/365/183	99.44%	>122 s	N/A	1
[40]		(activity recogn.)				Accelerometer, barometer				6	2.083333333	85.48%	N/A	N/A	5
[31]			×			GPS			Acessed URLs–browser history, phone calls, SMS	50	12	N/A	With 95% probability, the adversary will be locked out after 16 or fewer usages of the device	N/A	1
[41]						GPS				10	28	86.6%	30 minutes	N/A	4
[42]		×						Screen info		18	N/A	97.33%	N/A	2.03% FP, period unknown	-
[43]		×						Screen info		75	N/A	95.7%	0.648 s		7
[44]		×				Accelerometer				36	24	78.78%	30 s	3.97% FP, period unknown	Multiple
[32]			×			Location		Camera, screen info		48	60	65–95%	N/A	N/A	1 (70%)
[45]						Accelerometer, gyroscope	×	×	Smartwatch accelerometer	6	2	97.4%	N/A	1.12% FP, period unknown	1
[46]		×				Accelerometer, gyroscope		Screen info, audio		10	7	91.67	N/A	N/A (in the terms of this paper)	1
[11]			×			Accelerometer, gyroscope, cell ID	×	Screen info, audio		7	N/A	>99%	N/A	>60%	1
[47]		×				Accelerometer, gyroscope		Screen info		n.a.	n.a.	N/A	N/A	N/A [t]	Multiple
[48]		×						Screen info		80	n.a.	99.99%		99.99% [b]	-
[49]		×				Accelerometer, orientation		Screen info		104	N/A	0.31 EER	N/A	N/A	-
[50]		×						Screen info		25	n.a.	0.04 EER	N/A	N/A	1
[51]		×				Accelerometer, gyroscope and magnetometer		Camera		10	70	73%	N/A	1% FP, unknown period	-
[52]		(activity recogn.)				Accelerometer, pressure		Audio		30	N/A	94%	N/A	N/A	1
[53]		×				Proximity, accelerometer, gyroscope, magnetometer				16	N/A	96%	N/A	N/A	1
[12]			×	App	Battery	Location	Cell ID, global Rx/Tx data			7	21	72%	N/A	N/A	1
[54]		×		App CPU, transmitted data		Accelerometer and gyroscope	×	×	×	8	240	>99%	1,5 min (average)	1 false positive every 2 weeks	1
[32]			×	App		GPS	WiFi		Times an app is visited, text through key board, browser history	200	30	0.01 < EER < 0.05	N/A	N/A	1 (60%)
[33]			×	App		GPS	Cell ID, phone calls, SMS			71		95.83%	N/A	11.45% FP	-
[55]		×			Battery					645	28	60% (intermediate)	N/A	42% FP	1 (75%)
[56]		×				Accelerometer, gyroscope				24	14	96.3%	2.4 ms	7.6%	-
[34]			×		Battery	GPS, accelerometer, magnetometer	Phone call	Audio, light, screen info		15	3	0.25 < F1 score < 0.9	N/A	N/A	1 (80%)
[35]			×			Accelerometer, gyroscope			Involves smartwatches	35		98.1%	21 ms	0.9%	6
**OURS**			×		**Battery**		**Global Rx/Tx data**	**Audio, light**		**50**	**730**	**97.05%**	**150 s (battery average with ST=20)**	**Tunable with ST**	**Not needed**
